# Nanoemulsion of *Sideroxylon obtusifolium* as an Alternative to Combat Schistosomiasis

**DOI:** 10.3389/fpls.2022.853002

**Published:** 2022-05-26

**Authors:** Leonardo da Silva Rangel, Adriana Passos de Oliveira, Deborah Quintanilha Falcão, Marcelo Guerra Santos, Natalia Lindimar Von Ranke, Carlos Rangel Rodrigues, José Augusto Albuquerque dos Santos, Leandro Rocha, Robson Xavier Faria

**Affiliations:** ^1^Laboratório de Avaliação e Promoção da Saúde Ambiental, Instituto Oswaldo Cruz – Fiocruz, Rio de Janeiro, Brazil; ^2^Programa de Pós-Graduação em Ciências e Biotecnologia, Universidade Federal Fluminense, Niterói, Brazil; ^3^Faculdade de Farmácia, Universidade Federal do Rio de Janeiro, Rio de Janeiro, Brazil; ^4^Laboratório de Tecnologia de Produtos Naturais, Universidade Federal Fluminense, Niterói, Brazil; ^5^Departamento de Ciências, Faculdade de Formação de Professores, Universidade do Estado do Rio de Janeiro, Rio de Janeiro, Brazil

**Keywords:** nanoemulsion, molluscicide, *Biomphalaria*, *Sideroxylon obtusifolium*, plant extract

## Abstract

Schistosomiasis is caused by the intestinal parasite *Schistosoma mansoni*. Individuals are affected by schistosomiasis when they are exposed to aquatic environments contaminated with *Schistosoma cercariae* that emerged from the infected intermediate host mollusk of the genus *Biomphalaria*. The WHO recommends using molluscicidal products to reduce the snail population and disease transmission. The WHO encourages the search for alternative substances in schistosomiasis control. Natural products are seen as a promising alternative because they are abundant in countries where schistosomiasis is endemic and have many different substances in their extracts, impairing cases of resistance. Therefore, the nanoemulsion effect of a butanol-soluble fraction of *Sideroxylon obtusifolium* leaves was evaluated against three study points in the biological cycle of the disease, that is, adults and young *Biomphalaria glabrata*, spawning by the host mollusk, and infectious larvae of the parasite. Extract-SOB (butanol fraction) and nano-SOB (nanoemulsion) demonstrated promising activity in adult *B. glabrata* population control with an LC_50_ of 125.4 mg/L, an LC_90_ of 178.1 mg/L, an LC_50_ of 75.2 mg/L, and an LC_90_ of 97 mg/L. Nano-SOB presented greater potency against young *B. glabrata*, with an LC_90_ of 72.1 mg/L and an LC_50_ of 58.3 mg/L. Still, relevant activity against *S. mansoni* cercariae was eliminated in 4 h (LC_90_: 34.6 mg/L). Nano-SOB reduced viable spawning by approximately 30% at 178.1 and 97 mg/L. Referring to most substances in this extract, quercetin-3-rhamnosyl-(1-6)-galactoside and hyperoside may cause low environmental toxicity and human toxicity according to *in silico* analysis. Thus, nano-SOB is a promising agent to combat *B. glabrata* population growth and schistosomiasis transmission.

## Introduction

Schistosomiasis is an acute parasitic and chronic disease caused by worm trematodes of the genus *Schistosoma* and is transmitted to many snail types. This disease affects 78 countries, mainly in tropical and subtropical regions. Schistosomiasis is the second largest infectious parasitic disease in the world after malaria (WHO, 2020a). In 2019, the estimates showed that at least 236.6 million people needed treatment for schistosomiasis. At least 105.4 million people in the world were treated for schistosomiasis (WHO, 2020b).

Schistosomiasis is caused by the intestinal parasite *Schistosoma mansoni*, which uses mollusks of the genus *Biomphalaria* as an intermediate host, favoring the transmission cycle (World Health Organization, 1983). The individuals were affected by schistosomiasis when they were exposed to aquatic environments contaminated with *Schistosoma* cercariae that emerged from the infected intermediate host. Therefore, in the installation of the evolutionary cycle of *S. mansoni*, the presence of susceptible *Biomphalaria* is necessary. Furthermore, *Biomphalaria glabrata* is the most efficient intermediate host of *S. mansoni* due to a high degree of susceptibility of this parasite (Rey, 2010). Therefore, without a compatible mollusk, transmission is restricted (WHO, 2019a,b).

In combination with other interventions, the WHO recommends using molluscicidal products, with a strategy of focal or seasonal application in freshwater areas and their margins. Thus, the molluscicidal application reduces the transmission rate by reducing the mollusk population (BRASIL, 2014; WHO, 2019a,b).

Niclosamide (Bayluscide^®^, Bayer, Leverkusen, Germany) is a commercially available molluscicide recommended by the WHO (World Health Organization, 1983; BRASIL, 2014) for a large-scale use in schistosomiasis control programs. However, this drug causes toxicity to other organisms, and mollusk populations exhibit resistance to niclosamide^®^. Thus, the search for new drugs and substances to control the vector is necessary. Therefore, the WHO encourages the search for alternative substances to control schistosomiasis (Inobaya et al., 2014). Natural products are seen as a promising alternative because they are abundant in countries where schistosomiasis is endemic and have many different substances in their extracts, making resistance difficult (Cantanhede et al., 2010; Ding-Feng, 2010).

Numerous plant species have been evaluated, some of which have been recognized for their molluscicidal activity. However, there are no data in the literature on this activity with *Sideroxylon obtusifolium* (Humb. Ex Roem. & Schult.) T.D. Penn. (Sapotaceae).

*Sideroxylon obtusifolium*, also called “quixaba,” “sapotiaba,” and “ruptures gibbon” (Silva et al., 2012), is a medicinal tree species found in the Restinga and Caatinga areas. This species can reach up to 15 m. This plant is widely known and is used in popular medicine (anti-inflammatory and healing) and commercial herbal products (Albuquerque and Oliveira, 2007). In addition, *S. obtusifolium* possesses confirmed biological activities, such as antifungal (Pereira et al., 2016), antimicrobial, and anti-inflammatory (Aquino et al., 2016; Carnevale Neto et al., 2017) activities.

The species of the Sapotaceae family are characterized by the diversity of substances resulting from their secondary metabolisms, such as triterpenes, steroids, tannins, flavonoids, and polyphenols, in addition to alkaloids, carotenes, cyanogenic compounds, carbohydrates, and fatty acids (Beltrão, 2000; Oliveira et al., 2012, 2013). Some chemical classes of natural origin have been described to have biological activity, especially flavonoids. Therefore, developing stable carrier systems to increase aqueous dispersibility and bioavailability can reduce this inconvenience. In this context, nanotechnology presents a safe alternative capable of releasing hydrophobic active substances in an aqueous medium and allows stable and adequate systems to be obtained for therapeutic use. Among several options for drug nanocarriers, nanoemulsions (NEs) were chosen for this work (Donsì and Ferrar, 2016).

Most NEs are nanosized (20–200 nm range) oil-in-water droplet dispersions stabilized by surfactants (Sagalowicz and Leser, 2010; Jaiswal et al., 2015). The main advantages of these nanocarriers are enhanced solubility and bioavailability, improved pharmacological activity and stability, more sustained delivery, and protection from physical and chemical degradation (Campos et al., 2012).

Thus, we aimed to evaluate the effectiveness of a nanoemulsion produced with the extract-SOB of *S. obtusifolium* leaves, demonstrating this activity at three points in the biological cycle of the disease as follows: adults and young *B. glabrata*, spawning of the host mollusk, and infectious larvae of the parasite (*S. mansoni* cercariae).

## Methodology

### Collection, Identification, and Extract Preparation

The *S. obtusifolium* leaves were collected in Restinga de Jurubatiba National Park, located in the municipality of Carapebus in Rio de Janeiro, with the authorization for activities with scientific purpose number 13659-14 (SISBIO) and SISGEN authorization number A0D648D. Prof. Dr. Marcelo Guerra Santos identified and deposited the exsiccate (no. 2152) at the Herbarium of the Faculty of Teacher Education at the State University of Rio de Janeiro (RFFP).

### Extract Preparation

The collected leaves (600 g) were dried in an oven at 40°C with forced ventilation for 48 h. Once dried, this vegetable material was crushed in a hammer mill and was subjected to extraction by maceration at room temperature using 96° GL ethanol as a solvent. This procedure was followed by filtration and concentration in a rotary evaporator, thus obtaining the crude extract (149 g). Then, liquid/liquid partitions were performed for the previous purification of this ethanolic extract of the tris way: 112 g of this ethanolic extract was resuspended in 90% ethanol and was partitioned with solvents of increasing polarity: hexane (14.6 g), dichloromethane (7.6 g), ethylacetate (17.6 g), and finally butanol to obtain 10.0 g of extract-SOB (Oliveira et al., 2012). The extract-SOB was subsequently diluted with dimethylsulfoxide (DMSO) at 50%. Dilutions were made with distilled water to realize the experiments.

### Obtaining the Nanoemulsion – Oil/Water

Nanoemulsions were developed using an oil and a pair of surfactants previously selected, according to the methodology proposed by Azeem et al. (2009), with some adaptations by Falcão (2007) described as follows: purified water (60.0%), extract-SOB *of S. obtusifolium* (5.0%), transcutol^®^ (22.5%) as the oil phase solvent and an aqueous solution of PEG-40 with hydrogenated castor oil (6.25%) as the chosen surfactant, and propylene glycol as the cosurfactant (6.25%).

The O/W nanoemulsion was prepared by adding extract-SOB, PEG-40 hydrogenated castor oil and propylene glycol in Transcutol^®^ and dissolving it, under manual stirring, during heating in an electric plate until reaching 70–80°C. After reaching the desired temperature, the aqueous phase was gently poured for 30 s. Then, both phases were mixed by the inversion method under mechanical agitation using a four-blade propeller with 400 turns/min speed, by remaining under constant stirring of 400 turns/min at an ambient temperature for 10 min. Finally, the outside of the container was subjected to a cold- water bath. The O/W nanoemulsion obtained in this way, through a low-energy emulsification technique, occurs in the form of nanometric droplets, whose average diameter, according to the steps above, is between 1 and 200 nm.

### Stability Study

The stability of all emulsions was evaluated 1, 7, 15, 30, and 60 days after manipulation by macroscopic and microscopic analyses (color, visual aspect, phase separation, creaming, and sedimentation) (Falcão, 2007). All emulsions were maintained at room temperature (25 ± 2°C) in a screw-capped glass test tubes during this period.

### Molluscicidal Test

The molluscicidal assay was performed according to Santos et al.’s (2017) methodology adapted from the WHO (1965). In this methodology, 24-well plates are used, where mollusks of the species *B. glabrata* (free of *S. mansoni*) are placed individually in the wells of the plate, forming groups of three mollusks per concentration to be tested. The tests were performed in triplicate on different days, totaling nine mollusks per concentration at the end of the tests. The usual test volume is 2,000 μL of test solution for 10–12 mm *B. glabrata* mollusks, which are satisfactorily stored in this volume. In smaller mollusks, it is necessary to increase the volume so that there is no leakage of the liquid medium. The mollusks used were collected from the breeding tanks. The specimens were kept with dechlorinated water and fed fresh lettuce (*Lactuca sativa* L. 1758) at the Lauro Travassos Pavilion of the Oswaldo Cruz Institute in Rio de Janeiro.

Initially, the butanol fraction of *S. obtusifolium* (extract-SOB) was also analyzed at 50, 75, 100, 125, 150, 200, 250, and 300 mg/L in 2,000 μL. The test was performed in triplicate using groups of three *B. glabrata* mollusks of 10 to 12 mm in diameter.

Subsequently, the nanoemulsion (nano-SOB) was tested in triplicate using groups of three *B. glabrata* mollusks of 10 to 12 mm in diameter using 40, 60, 80, 100, 125, and 250 mg/L in a final volume of 2,000 μL.

Young *B. glabrata* mollusks were tested in two sizes, namely, 3–5 mm and 6–8 mm in diameter, at the concentrations mentioned above for nanoemulsion in triplicate using groups of three *B. glabrata* mollusks and a final volume in the test well of 2,700 μL. In this case, we increased the volume to ensure mollusk exposure in the nanoemulsion solution.

The negative controls used in all tests were distilled water and nanoemulsion without the active compound. The positive control was niclosamide^®^ (Bayluscide) at 2 mg/L. Snail mortality was observed after 1, 2, 3, 4, 5, 6, 24, and 48 h of treatment. The absence of snail retraction into their shells and hemolymph release were the criteria for death detection. The molluscicidal activity was calculated as a percentage (%) of the niclosamide effect.

### Cercaricidal Test

According to the 24-well plate methodology by Santos et al. (2017), in one well of a 24-well plate, 1 mL of the cercariae suspension was placed in contact with 1 mL of the nanoemulsion to obtain a volume of 2 mL per well, and 20 μL of the vital dye trypan blue 0.1% was added. The following concentrations were tested: 40, 60, 80, 100, 125, and 250 mg/L. Mortality was assessed every hour until the treatment was completed for 4 h using a stereoscope microscope with an 11.5 × magnification to observe the dead cercariae stained blue. The live cercariae did not present a color. The positive control was 1% niclosamide^®^ (NCL), and the negative controls were chlorinated water and nanoemulsion without the active compound.

### Ovicidal Test

*Biomphalaria glabrata* spawns are used with embryos from 24 to 72 h of life. The spawns were stored in the wells of a 24-well plate according to the methodology of Santos et al. (2017). We exposed 1 mL of nano-SOB, both in the concentration of CL_90_ obtained from adult *B. glabrata* tests. The positive control was 1% niclosamide^®^ (NCL), and the negative controls were chlorinated water and nanoemulsion without the active compound. The exposure was executed on day 0, the viable embryos were counted, and the viability was evaluated at 24 and 48 h. The mortality parameters were color change and/or condensed appearance of the embryo.

### *In silico* Assays

The prediction of the ecotoxicity profile of the major substances from the extract-SOB, namely, quercetin-3-rhamnosyl-(1-6)-galactoside and hyperoside, was performed by ADMET Predictor™ (version 9.5, Simulations Plus, Lancaster, CA, United States). The endpoints analyzed were bioconcentration, biodegradation, impact at three different trophic levels [*Tetrahymena pyriformis*, water flea (*Daphnia*), and fathead minnow], and effects due to interference with sex hormones (estrogen and androgen disruptors).

### Statistics

For statistical verification, linear regression analysis and two-way ANOVA were used, with a value of *p* < 0.0001, applying GraphPad Prism software (version 5), showing *R*^2^ > 0.800. The LC_50_ and LC_90_ values were calculated in Excel version “W” using the dispersion analysis formula.

## Results and Discussion

### Nanoemulsion

Several emulsions were prepared with extract-SOB. The optimized process parameters found in the preliminary study were applied as the concentration of the extract-SOB and the surfactant couple (data not shown). Nano-SOB also demonstrated satisfactory characteristics for nanoemulsions, with an average particle diameter of 21.2 nm and stability after 60 days (Table 1). Therefore, it was possible to evaluate the polydispersity and microscopic aspects of the parameters. Due to their characteristic size, they possess stability against sedimentation, creaming, flocculation, or coalescence. This long-term physical stability of nanoemulsions makes them unique, and they are sometimes referred to as “approaching thermodynamic stability” (Solans et al., 2005).

**TABLE 1:** Characteristics of the nanoemulsion.

Emulsion	Macroscopic aspect	Microscopic aspect	Emulsion viscosity and direction	Stability at 50°C after 60 days	Average particle diameter (nm)	Polydispersity
5% partition butanolic	Transparent	Optical void	Oil/Water	Stable	21.2 ± 0.7	0.215 ± 0.006

According to Oliveira et al. (2012), from extract-SOB, a total of four saponins and ten flavonol glycosides were isolated and identified. However, these substances are characterized by great structural diversity and need a stable carrier system to increase aqueous dispersibility and bioavailability, which was developed in this study.

The major substances in this extract are quercetin-3-rhamnosyl-(1-6)-galactoside (47.2 mg; tR 16.0 min), which was purified by preparative HPLC from Fr. 117–128 (0.300 g) with a gradient of (MeCN/0.1% aq. HCOOH 40:60–50:50 in 40 min), and hyperoside (619.5 mg; tR 17.0 min), which was purified by preparative HPLC from Fr. 141–160 (0.400 g) with a gradient of (MeCN/0.1% aq. HCOOH 40:60–50:50 in 40 min) according to the work of Oliveira et al. (2012), who used a fraction of the material used in the present study.

Hyperoside is a flavonol glycoside, also known as quercetin-3-O-D-galactoside, in various vegetables and fruits (Wu et al., 2007; Hosseinimehr et al., 2008). Identified in different plant products, it has become important due to its diverse potent pharmacological activities, including anti-inflammatory, antithrombotic, antidiabetic, antiviral, antifungal, hepatoprotective, and antioxidant protective effects (Raza et al., 2017).

### Biological Activity Tests

According to documents from the WHO (World Health Organization, 1983; WHO, 2017, 2019a), a molluscicide originating from the plant is considered promising when this substance reaches mortality in 90% of the population at a concentration of 100 mg/L (LC_90_) in 48 h.

**FIGURE 1:**
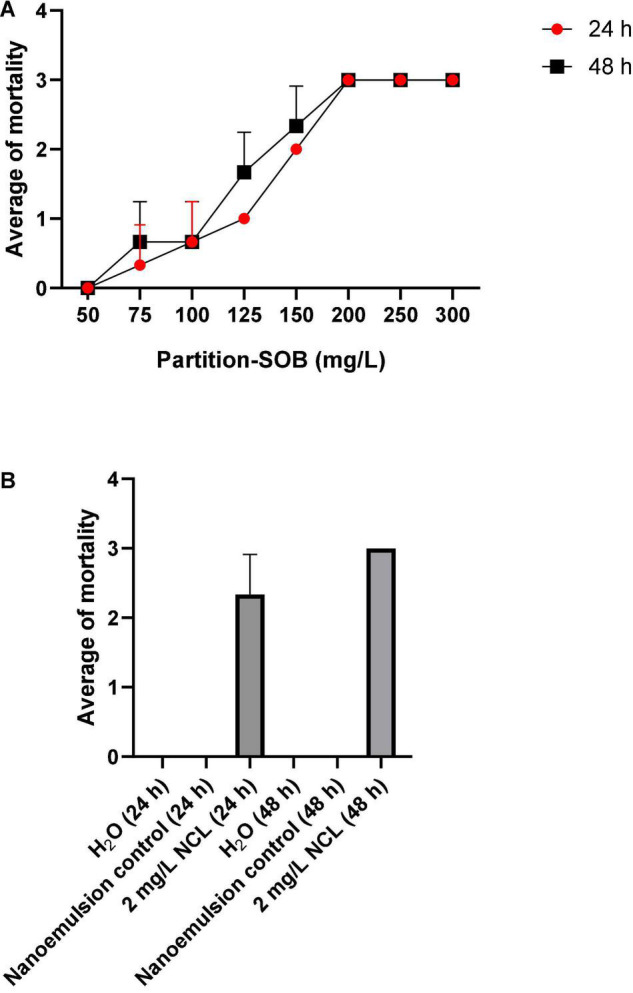
The molluscicidal test on adult *Biomphalaria glabrata* for 48 h using extract-SOB **(A,B)** The test was performed in triplicate on different days using 27 specimens/triplicate. These data are expressed as the mean ± SD. Using the linear regression analysis, the value of *R*^2^ is determined as 1.00.

We tested the butanol-soluble fraction derived from a crude ethanol extract of *S. obtusifolium* leaves (extract-SOB) against adult *B. glabrata* snails (10–12 mm in length). The results showed maximal activity of extract-SOB in the first 24 h with a maximal concentration of 200 mg/L, maintaining the result in 48 h. The treatment with extract-SOB promoted the LC_50_ of 125.4 mg/L and the LC_90_ of 178.1 mg/L (Figure 1A), in this case exceeding the WHO-recommended value (100 mg/L). On the other hand, niclosamide^®^ (NCL) treatment at 2 mg/L caused 100% death after 24 h, and H_2_O or nanoemulsion control did not cause toxic effects (Figure 1B).

**FIGURE 2:**
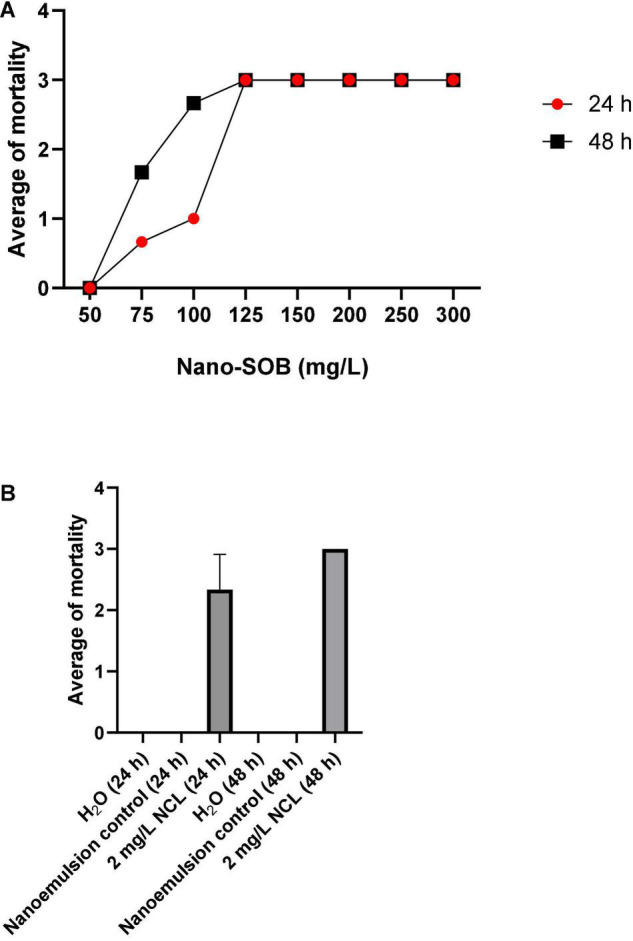
The molluscicidal test on adult *Biomphalaria glabrata* for 48 h using nano-SOB **(A,B)**. The test was performed in triplicate on different days using 27 specimens/triplicate. These data are expressed as the mean ± SD. Using the linear regression analysis, the value of *R*^2^ is determined as 0.852, with a *P* value of 0.0074.

Nano-SOB enhances the aqueous dispersibility and extracts yield as an optimization method. The molluscicidal test of nano-SOB showed maximal activity at 125 mg/L after 24 and 48 h of analysis. The lethal concentrations obtained were LC_50_ = 75.2 mg/L and LC_90_ = 97 mg/L when tested on adult *B. glabrata* (Figure 2A). Niclosamide^®^ (NCL) treatment at 2 mg/L caused 85% mortality after 24 h and 100% mortality after 48 h. The H_2_O or nanoemulsion control did not cause a toxic effect (Figure 2B).

**FIGURE 3:**
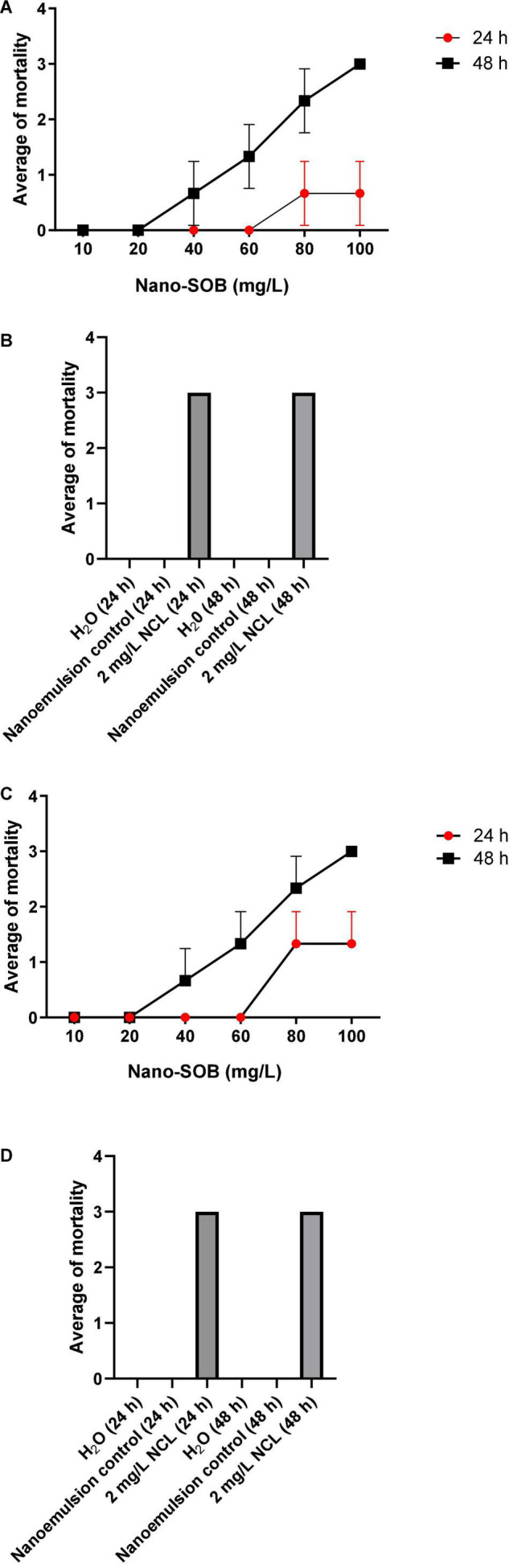
The molluscicidal tests on young *Biomphalaria glabrata* 6–8 mm **(A)** and 3–5 mm **(C)** for 48 h using nano-SOB **(B,D)**. Niclosamide^®^ (NCL) and the nanoemulsion control did not cause toxicity. The test was performed in triplicate on different days using 27 specimens/triplicate. These data are expressed as the mean ± SD. Using the linear regression analysis, the value of *R*^2^ is determined as 1.00, with a *P-*value of 0.0022.

**FIGURE 4:**
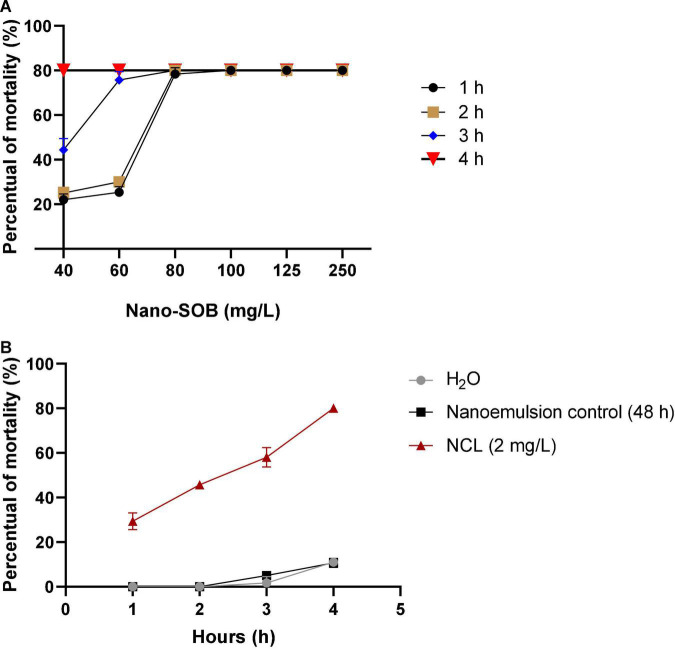
Nano-SOB activity against *Schistosoma mansoni* cercariae for 4 h **(A,B)**. The test was carried out in triplicate on different days using a range of 80 cercariae per well during the testing of the samples. These data are expressed as the mean ± SD. Using the two-way ANOVA test, the value of *R*^2^ is determined as 0.8660, with a *P-*value of < 0.0001.

**FIGURE 5:**
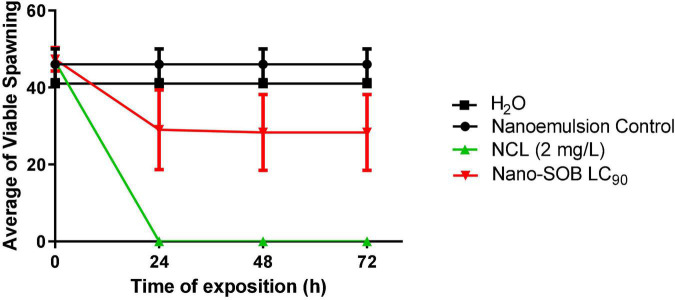
Effect of nano-SOB on the spawning of *Biomphalaria glabrata* in the period from 24 to 72 h of exposure. The test was repeated three times in triplicate. A range of 40–45 spawns was used per well. These data are expressed as the mean ± SD. Using the two-way ANOVA test, the value of *R*^2^ is determined as 0.5820, with a *P* value of 0.0077.

Nano-SOB was evaluated against young *B. glabrata* of two sizes (3–5 and 4–6 mm). The results were similar after 48 h of exposure, with a maximal activity at 100 mg/L. Interestingly, nano-SOB treatment was more potent in causing a toxic effect in both early stages when compared with adult snails. The LC_90_ value was 72.1 mg/L and the LC_90_ was 58.3 mg/L for *B. glabrata* from 6 – 8 mm (Figure 3A) and 3 – 5 mm (Figure 3C). On the other hand, niclosamide^®^ (NCL) treatment at 2 mg/L caused 100% mortality after 24 h, and H_2_O or nanoemulsion control did not cause toxic effects (Figures 3B,D).

Nano-SOB exhibited molluscicidal activity against adult and young snails. Thus, we investigated the effect on cercariae released from infected *B. glabrata*. We treated cercariae for 4 h and analyzed viability each hour. Niclosamide^®^ treatment at 2 mg/L caused 80% mortality after 4 h. Treatment for 1, 2, and 3 h produced toxicities of 30, 46, and 60%, respectively. H_2_O or nanoemulsion control caused a discrete toxic effect of 10% in 4 h. Thus, nano-SOB exhibited a dose-dependent effect (Figure 4). The nano-SOB concentration of 80 mg/L promoted a maximal activity of 80% after 1 h of exposure. The concentration of 40 mg/L reached this same maximal effect after 4 h of exposure. The LC_50_ value calculated for the nano-SOB treatment after 4 h was 20.09 mg/L and the LC_90_ value was 23.63 mg/L (Table 2).

**TABLE 2:** Kinetics of nano-SOB toxicity on *Schistosoma mansoni* cercariae.

Time of exposition (h)	Nano-SOB LC_50_ (mg/L)	Nano-SOB LC_90_ (mg/L)
1	57.49	80
2	55.11	77.79
3	36.60	55.14
4	20.09	23.73

Other studies, such as Vinaud et al. (2005), found maximal cercaricidal activity in 1 h at 20 to 200 mg/L concentrations.

Another possibility of controlling the snail population is the control of spawning to influence the mollusk reproductive cycle.

The nano-SOB LC_90_ concentration calculated for adult *B. glabrata* was determined after exposure to embryos for 24 and 72 h (Figure 5). Treatment with 2 mg/L NCL caused a complete reduction of viable spawning in 24 h. The nanoemulsion control did not cause toxicity. Nano-SOB treatment diminished the viable spawning number. However, the nano-SOB effect was poor when compared with that of NCL.

**FIGURE 6:**
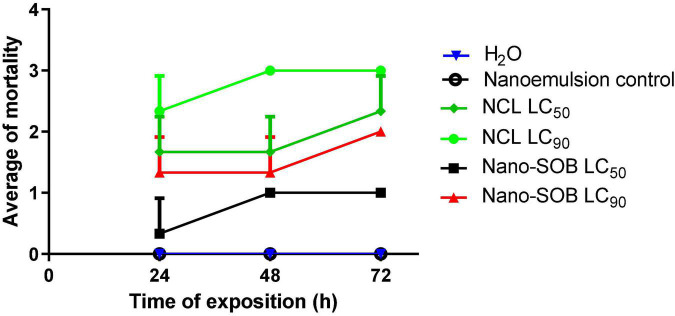
Mortality kinetics of *Melanoides sp.* Tests were performed in triplicate on three different days. These data are expressed as the mean ± SD. Using the two-way ANOVA test, the value of *R*^2^ is determined as 0.630, with a *P-*value of < 0.0080.

Luna et al. (2005) found 100% mortality of spawning at 100 mg/L *Caesalpinia echinata* (Pau-Brasil). Nano-SOB, tested in the present study, reduces viable spawning within 48 h of exposure. We tested nano-SOB on another snail species, *Melanoides sp*., related to schistosomiasis transmission and environmental toxicity. The LC_50_ and LC_90_ values of nano-SOB were tested for 72 h (LC_50_: 75.2 mg/L and LC_90_: 97 mg/L). The NCL LC_50_ and LC_90_ values were obtained from Giovanelli et al. (2002). NCL LC_90_ promoted maximal *Melanoides sp*. toxicity after 48 h of exposure. The nanoformulation control did not cause a toxic effect in this snail (Figure 6). The LC_50_ and LC_90_ values of nano-SOB exhibited a moderate toxic effect on this species compared with NCL. This result could indicate a possible selectivity of the nanoformulation according to the species studied and indicate a possible environmental tolerance compared with NCL.

Evaluation of the environmental effects caused by commercial chemicals may prevent accidental toxic effects on aquatic species. Therefore, the chemical industry, pharmaceutical companies, and government agencies have established standard experimental protocols to test chemicals for their toxic potential (Zhu et al., 2008).

**SCHEME 1:**
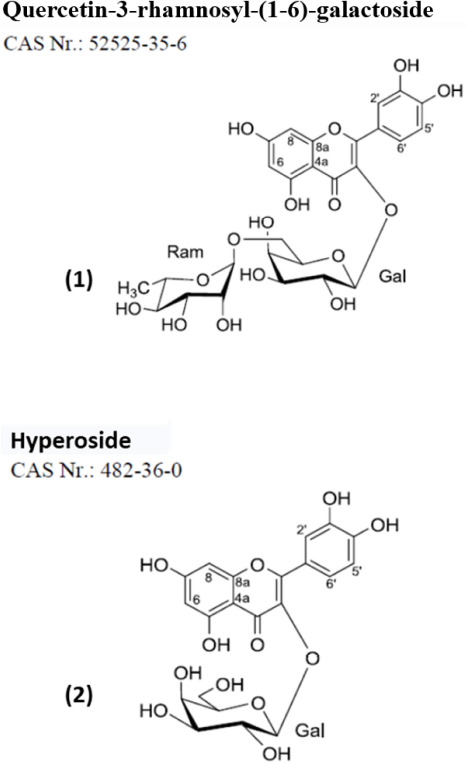
The 2D structural representation of (1) quercetin-3-rhamnosyl-(1-6)-galactoside and (2) hyperoside.

Although experimental protocols for toxicity testing have been developed for many years, computational chemical toxicology is a viable approach to reduce the number of efforts and the cost of experimental toxicity assessment. In this regard, we used the ADMET Predictor (Simulation Plus) to predict the environmental effects caused by the majority of substances from the extract-SOB. Thus, we evaluated the ecotoxicity of (1) quercetin-3-rhamnosyl-(1-6)-galactoside and (2) hyperoside (Scheme 1).

The bioconcentration factor (BCF) is the ratio of the chemical concentration in biota to that in water at a steady state (Hamelink and Spacie, 1977). Environmentally, the BCF describes the accumulation of pollutants partitioning from the aqueous phase into an organic phase (typically fish) and does not include uptake due to diet. The BCF has no units, as shown in equation (a):


(a) BCF=[Conc.inorganisms]/[Conc.intheenvironment].

Some reference values have been defined for BCF risk assessment. A substance with a BCF greater than 2,000 will be regarded as bioaccumulative for EU REACH. A substance with a BCF greater than 5,000 will be regarded as very bioaccumulative. In the United States, a substance is considered not bioaccumulated for a BCF value less than 1,000, bioaccumulated for a BCF value from 1,000 to 5,000, and very bioaccumulated for a BCF value greater than 5,000.

From the reference values cited, all investigated compounds predicted had a low bioaccumulative risk. Interestingly, none of the tested compounds presented an alarming bioaccumulative risk (greater than 5,000) (Table 3).

**TABLE 3:** Results of the compound ecotoxicity.

Compounds	BCF	BOD	Aquatic toxicity			Endocrine receptor binding	
			Th_pyr_pIGC_50_	Daphnia_LC_50_	Minnow_LC_50_	Andro_Filter	Estro_Filter
Quercetin-3-rhamnosyl-(1-6)-galactoside	0.422	No	–1.292	32.942	1365.521	Toxic	Toxic
Hyperoside	1.015	No	–0.557	54.841	350.178	Toxic	Non-toxic

*The endpoints evaluated for each compound are defined as follows: BCF, bioconcentration factor; BOD, biodegradation – categorizing the compounds as positive (readily biodegradable) if % biodegradation is greater than or equal to 60% and as negative otherwise; Th_pyr_pIGC_50_, concentration (mM) of toxicant needed to inhibit growth by 50% (IGC_50_) of Tetrahymena pyriformis; Daphnia_LC_50_, concentration (mg/L) of compound required to kill 50% of a D. magna population; Minnow_LC_50_, concentration (mg/L) of a compound that kills 50% of a population of minnows; Andro_Filter and Estro_Filter assess a compound’s likelihood of binding to the androgen and estrogen receptors, respectively.*

Such findings agree with the compound physical–chemical characteristics. The compounds that presented a higher risk of being bioaccumulative were those with lower water solubility.

Biodegradation (BOD) of chemicals in the environment is becoming increasingly important. For the biodegradation assessment, the compound of interest is mixed with sludge from several geographical locations; then, oxygen consumption is measured for the specified period. Biological oxygen demand (BOD) is calculated from equation (b):


(b) $$B\,O\,D=\frac{\left[O_{2}\,u\,p\,t\,a\,k\,e\,b\,y\,t\,e\,s\,t\,s\,u\,b\,s\,t\,a\,n\,c\,e\right]-\left[O_{2}\,u\,p\,t\,a\,k\,e\,b\,y\,b\,l\,a\,n\,k\right]}{\left[a\,m\,o\,u\,n\,t\,o\,f\,t\,e\,s\,t\,s\,u\,b\,s\,t\,a\,n\,c\,e\right]}$$

The percentage biodegradation is obtained from equation (c):


(c) $$\%biodegradation=\frac{B\,O\,D}{T\,h\,O\,D}*100\%$$

ThOD represents the amount of oxygen consumed in converting all the compounds to carbon dioxide (CO_2_), i.e., the theoretical oxygen demand. The ADMET predictor categorizes the compounds as positive (readily biodegradable) if % biodegradation is greater than or equal to 60% and as negative otherwise. Thus, the model returns a “yes” if the compound is readily biodegradable or “No” if not.

All the compounds tested are predicted to be non-biodegradable. Therefore, attention must be paid to the amount of waste generated in the environment and its potential harm (Table 3).

Aquatic toxicity can also be measured as its potential to inhibit the growth of living aquatic beings. Therefore, the ADMET predictor provides three models to investigate this characteristic at different trophic levels [*Tetrahymena pyriformis*, water flea (Daphnia), and fathead minnow].

The model that evaluates *T. pyriformis* growth inhibition predicts the concentration (mM) of toxicant needed to inhibit growth by 50% (IGC_50_) in the protozoan after approximately 40 h of exposure (eight to nine cell cycles in the control group) at 27°C.

The *T. pyriformis* model showed that 20% of the most toxic compounds presented pIGC_50_ values less than approximately -1.0. Therefore, quercetin and hyperoside presented a higher potential to inhibit *T. pyriformis* growth. Water fleas (*Daphnia magna*) occupy the next rung up on the aquatic food chain from Tetrahymena. Daphnia_LC_50_ predicts the concentration of compounds required to kill 50% of a *D. magna* population within 48 h. The data for this study were obtained from the EPA’s ECOTOX database.

The developed *D. magna* model showed that 20% of the most toxic compounds presented an LC_50_ of less than approximately 3.0. Therefore, the compounds, quercetin and hyperoside, did not present potential aquatic toxicity.

The third trophic level investigated was the effect of the compound on *Pimephales promelas*. The ADMET predictor model was developed based on the database created by the Mid-Continent Ecology Division of the U.S. Environmental Protection Agency testing industrial compounds for lethal effects on the fathead minnow (*P. promelas*). The experimental protocol was used to determine the concentration of a compound that killed 50% of a population of minnows after an exposure time of 96 h.

The *P. promelas* model showed that approximately 20% of the most toxic compounds presented an IGC_50_ less than 2.0. Therefore, quercetin and hyperoside did not present potential aquatic toxicity.

The potential environmental damage caused by exposure to endocrine receptor binding disruptors has gone unappreciated until relatively recently. The compounds compete for binding with estrogenic or androgenic sex hormones, blocking the timely transmission of standard hormonal signals or producing them in a few cases.

ADMET predictor models of endocrine receptor binding are used to assess a compound’s likelihood of binding to the estrogen/androgen receptor. Estro_Filter and Andro_Filter are straightforward predictions of whether the molecule will have an appreciable affinity for the estrogen or androgen receptor at all. A negative (non-toxic) classification indicates that the compound is unlikely to cause endocrine disruption by binding to the receptor. In contrast, a positive (toxic) indicates that the compound is likely to bind detectably to the receptor.

Regarding the estrogen receptor, hyperoside is predicted to have a non-toxic effect compared with quercetin. On the other hand, quercetin and hyperoside have the potential to interact with the androgen receptor. Therefore, these compounds risk adverse health outcomes, including cancer, reproductive impairment, cognitive deficits, and obesity.

## Conclusion

Nano-SOB ameliorated the extract-SOB molluscicidal activity on the population of *B. glabrata.* This biotechnological strategy rescued extract-SOB activity with an LC_90_ value in the range recommended by the WHO. Nano-SOB also acted on young *B. glabrata* mollusks with similar potency to adult *B. glabrata*. Additionally, nano-SOB exhibited notable activity against the cercariae of *S. mansoni*, where, after 4 h, all the cercariae were eliminated. Furthermore, extract-SOB and nano-SOB were not effective in reducing viable spawning. Concerning ambient toxicity, nano-SOB was moderately toxic to *Melanoides sp*. Thus, nano-SOB proved to be an excellent alternative in controlling and fighting against schistosomiasis, acting in several disease transmission cycle phases. Additionally, quercetin-3-rhamnosyl-(1-6)-galactoside and hyperoside are biodegradable and pose a low risk of environmental accumulation and aquatic toxicity. However, these substances pose a risk for human manipulation.

## Data Availability Statement

The raw data supporting the conclusions of this article will be made available by the authors, without undue reservation.

## Author Contributions

APO, DQF, and LR performed the experiments based on chemical isolation of the essential oil and nanoemulsion. LSR performed the experiments based on biological assays. NLVR and CRR performed the experiments based on *in silico* assays. MGS identified and deposited the exsiccate. RXF, JAAS, and LR wrote and supervised the manuscript. All authors contributed to the article and approved the submitted version.

## Conflict of Interest

The authors declare that the research was conducted in the absence of any commercial or financial relationships that could be construed as a potential conflict of interest.

## Publisher’s Note

All claims expressed in this article are solely those of the authors and do not necessarily represent those of their affiliated organizations, or those of the publisher, the editors and the reviewers. Any product that may be evaluated in this article, or claim that may be made by its manufacturer, is not guaranteed or endorsed by the publisher.
